# Collective Re-Storying of Mentee and Mentor Experiences in a Cancer Research Education Program for American Indian and Alaska Native Students

**DOI:** 10.15695/jstem/v5i2.08

**Published:** 2022-08-31

**Authors:** Kiana Borengasser, Aislinn C. Rookwood, Joyce C. Solheim, Maurice Godfrey, K. Taraszka Hastings, Keyonna M. King, Hannah Robbins, Mariah Abney, Rudy Smith, Liliana Tamayo, Regina E. Idoate

**Affiliations:** 1 Kamealohaokekaiakea, LLC, Waianae, HI;; 2 Department of Health Promotion, University of Nebraska Medical Center, Omaha, NE;; 3 Eppley Institute and the Fred and Pamela Buffett Cancer Center, University of Nebraska Medical Center, Omaha, NE;; 4 Munroe-Meyer Institute, University of Nebraska Medical Center, Omaha, NE;; 5 Department of Basic Medical Sciences, College of Medicine Phoenix, University of Arizona, Phoenix, AZ;; 6 University of Arizona Cancer Center, Tucson, AZ;; 7 Phoenix Veterans Affairs Health Care System, Phoenix, AZ;; 8 Arizona Department of Health Services, Phoenix, AZ;; 9 University of Nebraska at Omaha, Omaha, NE;; 10 Arizona State University, Tempe, AZ;; 11 Boys and Girls Clubs of the Midlands, Omaha, NE

**Keywords:** Mentor, Mentee, Cancer Research, Health Professions, Undergraduate, High School, Native American, American Indian, Alaska Native, Indigenous, Internship, Mentorship

## Abstract

The Youth Enjoy Science program at the University of Nebraska Medical Center has engaged American Indian/Alaska Native youth in mentored cancer research internships from 2017 to 2022. The primary purpose of this study was to examine mentor and mentee lived experiences of participation in Youth Enjoy Science research education internships and to provide insights that can inform mentorship practices in research education programs for American Indians/Alaska Natives. We conducted semi-structured interviews with current and former Youth Enjoy Science mentees (n=8) and mentors (n=8). Following a narrative inquiry research approach, we analyzed interview transcripts and collectively re-storied interview data. Participants described program characters, settings, problems, actions to address the problems identified, and resolutions that led to various recommendations for ways to raise contextual awareness between mentees and mentors.

## INTRODUCTION

American Indian and Alaska Native (AI/AN) populations experience significant health disparities (Indian Health Service [IHS], 2019; Kruse et al., 2022; Villarroel et al., 2020; Zuckerman et al., 2004). The National Cancer Institute (NCI) has recognized increasing representation of AI/ANs in the health science and research fields as one approach to addressing these health disparities. In 2019, approximately 1.7% of the US population identified as AI/AN, yet only 0.2% of total full-time faculty members at U.S. medical schools and teaching hospitals identified as AI/AN (Association of American of Medical Colleges [AAMC], 2019). Although approximately 41% of first-time, full-time AI/AN students attending four-year institutions graduated with a bachelor’s degree in 2019, only 14% of those were in a science, technology, engineering, or mathematics (STEM) field (compared to a 21% average for other racial/ethnic groups) (Dean, 2021; National Cancer for Education Statistics, 2022). Moreover, only 0.4% of doctoral degrees in the sciences and engineering were earned by AI/ANs in 2019 (National Center for Science and Engineering Statistics, 2019).

The Association of American Indian Physicians (2018) has documented a lack of AI/AN exposure to health professionals and a scarcity of faculty who can provide culturally relevant mentorship and support (AAMC and Association of American Indian Physicians, 2018). Moreover, Indigenous researcher, Linda Tuhiwai Smith (Ngāti Awa and Ngāti Porou, Māori), suggests that the word ‘research,’ among AI/ANs “stirs up silence, it conjures up bad memories, it raises a smile that is knowing and distrustful” (Smith, 2012, p. 1). Recognizing these barriers, the NCI-funded Youth Enjoy Science program at the University of Nebraska Medical Center (UNMC-YES) aims to strengthen trust in research and to increase AI/AN representation in health science and research fields through cancer research mentorships.

In the UNMC-YES program, the research training component engages students from middle school through undergraduate in research experiences such as secondary school-based cancer research club activities, medical center-based summer cancer research camps, and medical center-based paid cancer research internships. Students who meet the following criteria are eligible to participate in UNMC-YES cancer research internships: 1) they have current high school and undergraduate student status, 2) they self-identify as an AI/AN, and 3) they express an interest in health professions and/or STEM. A diverse spectrum of inter-tribal AI/AN students participate in internships, from students who reside in urban areas near UNMC-YES campuses to students who reside in rural areas and/or Tribal reservations, from STEM majors to art majors, and from students with a long-standing interest in pursuing a career in research to students with no initial interest. Mentees enter the program by applying to posted job listings and are selected based on the program’s ability to match mentor and mentee interests and location. The UNMC-YES program’s dependence on annual funding renewal makes intern terms of appointment limited to no more than one year, but the program aims to retain interns for a minimum of two years and has renewed appointments for every intern with an interest in continuing. The length in which mentors and mentees participate in mentorships varies widely, ranging from between several months to more than four years.

UNMC-YES partners with biomedical and public health researchers, university faculty, schoolteachers as well as AI/AN Elders and community members to provide AI/AN students with culturally appropriate cancer research experiences that acknowledge the students’ diverse backgrounds, knowledge, and interests. Mentors are required to have at least one year of experience in either biomedical or public health research. AI/AN students are paired with AI/AN and non-AI/AN mentors who engage them in research experiences through technology, wet lab techniques, community-based participatory research and/or Indigenous research methods. Internship sites are at the UNMC Eppley Institute, the University of Arizona College of Medicine Phoenix, or the UNMC College of Public Health. Each site has a program coordinator that supervises interns. Some coordinators also serve as mentors.

Nebraska is home to six tribes and represents the ancestral homelands for many Indigenous peoples including the Umó^n^Ho^n^ (Omaha), Ponca, Santee, Ho-Chunk Winnebago, Lakȟóta, Pawnee, Cheyenne, Arapaho, Meskwaki, Otoe-Missouria, Ioway, Kaw, Wichita, Kickapoo, and Delaware Nations (Swanton, 1952). AI/ANs comprise 1.5% of the Nebraska state population (U.S. Census Bureau, 2021). The Nebraska urban AI/AN population is representative of over 100 different Tribal nations from across the IHS Great Plains region and beyond (Robbins et al., 2019). Since 2017, a total of 21 AI/AN mentees have worked with mentors as UNMC-YES cancer research interns at the Eppley Institute and College of Public Health. These mentees identify as from the following Indigenous nations: ᏣᎳᎩᎯ ᎠᏴᎵ(Cherokee Nation), Oglala Lakȟóta (Lakota), Osage Nation, Siċaŋġu Lakȟóta (Lakota), Ho-Chunk Winnebago Tribe of Nebraska, Eastern Band of Cherokee, Crow Agency, Hopi, Cheyenne River Sioux, Umó^n^Ho^n^ (Omaha Nation), Santee Sioux, Keetoowah Cherokee, San Carlos Apache, Menominee, and Creek Sioux. Here, we used the Indigenous languages of the authors, including their syllabary as a sign of respect and support for the revitalization of Indigenous languages (Younging, 2018).

Arizona is home to 22 tribes and represents the ancestral homelands for many Indigenous peoples, including the Akimel O’odham, Pee Posh, O’odham and the Yaqui Indian Communities (Swanton, 1952). AI/ANs comprise 5.3% of the Arizona state population (U.S. Census Bureau, 2021). The Arizonan urban AI/AN population includes representatives of nations from across the Southwest region and throughout the U.S. Since inception of the program, a total of four AI/AN mentees have worked with mentors as UNMC-YES program cancer research interns at the University of Arizona College of Medicine Phoenix. These mentees identified as: ᏣᎳᎩᎯ (Cherokee), San Carlos Apache, Diné (Navajo) and Acoma Pueblo/Diné (Navajo). Here again, we included Indigenous language and syllabary to recognize the languages of our Indigenous authors (Younging, 2018).

There are few studies that capture the lived experiences of mentors and/or mentees in health research education programs that serve AI/AN populations (Jones et al., 2019; Lee et al., 2018; McMahon et al., 2019; Segrest et al., 2010). The primary purpose of this study was to examine mentor and mentee experiences of participation in the UNMC-YES cancer research internships to inform efforts to enhance AI/AN health research education in the U.S.

## METHODS

The five researchers who developed the study design identified as female, were affiliated with the UNMC-YES program, and had experience working in public health (three were current public health scientists); four of the five also had experience working in biomedical sciences (two were current lab scientists). Over 50% of our overall research team (authors) identified as Indigenous. The research team included UNMC-YES principal investigators (MPIs), program manager, external evaluator, and current and former interns.

### Participants.

Researchers identified 14 mentors and 25 mentees who participated in UNMC-YES program cancer research internships between 2017 and 2021. In 2021, we purposively recruited participants from this group under the assumption that the sample would be representative of the greater population of Indigenous health sciences mentees and their mentors. Mentee eligibility for participation included: a minimum of sixteen years of age and self-identification as an AI/AN with experience as a current or former cancer research intern in the UNMC-YES program cancer research internship program. Mentor eligibility included engagement in cancer research studies, a minimum of 19 years of age and self-identification as a mentor with at minimum one year of experience and expertise in recruiting, retaining, and/or mentoring students in the UNMC-YES research education program. All 14 mentors and 25 mentees from the recruitment pool were formally invited via email to participate in an interview. One mentee declined to participate. Six mentors and sixteen mentees did not respond to the invitation. An equal number of mentors (n=8) and mentees (n=8) responded positively to the invitation and gave informed written consent. The sample size of 16 was sufficient to reach saturation to the point of repetition (Sandelowski, 1995). Participation was strictly voluntary, and participants were ensured that deciding not to participate in this research study would not affect their relationship with study personnel or UNMC-YES. Mentee participants were provided a $50 gift card for participation. This study was reviewed and approved by the UNMC Institutional Review Board (IRB).

The mentors interviewed included faculty members, post-doctoral researchers, and graduate assistants who had worked with YES interns on cancer research projects. Only one of the eight mentors interviewed identified as AI/AN and seven, although not Indigenous, represented other diverse backgrounds. Some mentors specialized in community-based, public health cancer research (n=2) while others specialized in lab-based, biomedical cancer research (n=6). Four of the mentors identified as faculty members, two identified as postdocs, and two as graduate students. The mentees interviewed included students who participated in biomedical internships (n=2), students who participated in public health internships (n=2) and students who participated in both biomedical and public health internships (n=4) in the UNMC-YES program. Proportionate to the overall UNMC-YES intern demographics, mentee participants included two high school students and six undergraduate students. At the time of the interviews, two of the former undergraduate YES cancer research interns had graduated and held a bachelor’s degree.

### Interviews.

Mentors and mentees were interviewed by one of three members of the research team, which included two UNMC-YES program mentors and the UNMC-YES external program evaluator. One of the two program mentors was an Indigenous public health researcher with experience in community-based research, and the other was a non-Indigenous researcher with experience in both lab-based and community-based research. The UNMC-YES program external evaluator identified as Indigenous with experience in both lab-based and community-based research. To eliminate bias, no mentees were interviewed by their current or previous mentors. All interviews followed a semi-structured interview protocol. The interview guide consisted of approximately 20 questions, developed by UNMC-YES mentors and mentees, and were informed by both lived experience and scholarly literature (Murry et al., 2021; Liaw et al., 2016; Walters and Simoni, 2009). Interviews lasted approximately 45 to 60 minutes. Questions asked to mentors included: “How would you describe your role as a mentor to this intern student?,” “Is there anything that you do in your role as a mentor that specifically acknowledges Native American culture?,” and “How would you describe your experience of being a mentor with YES interns?” Questions asked to mentees included: “What are your thoughts about pursuing a research career?,” “How would you describe your internship experience in terms of communication of cultural knowledge between you and your mentor?,” and “How would you describe your overall experience with mentors in the YES program?”. All interviews were audio recorded and transcribed verbatim. Participant names and identifying information were removed from transcribed data to achieve anonymity.

### Analysis.

This qualitative study utilized a narrative inquiry research design that centered on storytelling, a traditional and culturally acceptable approach to communication and learning among AI/ANs (Cajete, 2005; Cajete, 2008; Iseke, 2013). Working from a decolonizing lens, we considered individual narratives in relation to one another to form a collective story that recognized a “Native collective consciousness” (Denzin et al., 2008, p. 310). Transcribed interview data were analyzed by the same three members of the research team who conducted the interviews. The research team employed a deductive-inductive restorying data analysis approach, deductively coding mentor and mentee experiences into narrative elements (characters, setting, problems, actions, and resolutions) and, subsequently, inductively coding common threads, resonant metaphors, and patterns of expression that demonstrated emergent themes from the data that was coded into each of the narrative elements (Ollerenshaw and Creswell, 2002; Creswell and Poth, 2016). Analysis involved reading and rereading the data interspersed with ongoing iterative discussions. See Table 1 for further description of what was included in each of the narrative elements.

Development of the re-storied narrative involved community member-checking (with four UNMC-YES mentees and four UNMC-YES mentors) to address potential bias that the researchers’ views and beliefs may have had upon the analysis (Fusch and Ness, 2015). Mentees and mentors discussed the accuracy and resonance of the data and findings with the research team and the research team modified interpretations accordingly. Following Indigenous methodologies, we sought to privilege Indigenous voices (Bessarab and Ng’Andu, 2010; Smith, 2012) and, thus, integrated Indigenous teachings, extensive mentee participant quotes and precise language into both the data analysis and dissemination processes.

## RESULTS

We report our findings according to the story elements from a problem-solution narrative structure: characters, settings, problems, actions, and resolutions (see Table 1).

### Characters: A Diverse Circle of Relatives.

The relationships involved in the UNMC-YES program’s health research mentorship reflects the Lakȟóta teaching, Mitákuye Oyás’iŋ. This Lakȟóta teaching translates roughly to “all my relatives”, meaning that no matter our differences we are all part of a bigger picture and what happens to one person can have a lasting impact on many people. UNMC-YES mentorship was not just a relationship between two people (mentee and mentor) but rather a relationship among many peoples, including AI/AN Elders, AI/AN community members, family members, program personnel, principal investigators, post-doctoral researchers, graduate assistants, lab technicians, and research interns. As one mentee understood it, “I’m basically there to support the grad student that I mentor with, and that grad student is there to support the principal investigator there. And so that’s kind of like how I fit into the lab.” The mentorship program involved multiple persons with unique roles and diverse personalities, backgrounds, attitudes, styles, and perspectives.

Mentors described mentees as “very curious,” “very hard-working,” and “really wonderful people… with a commitment to what they’re doing.” One mentor said, “I really feel like [the mentees] have developed a lot and in some ways I think the students are already pretty clear of what their research interests are and have a lot of innate skills that make them really great researchers to begin with.” As mentors noted, some of “their (mentees’) overall biology experience [was] limited” and/or they were “starting from a very low scientific research knowledge.” One mentor shared, “we had to really backtrack far back in because that level of technical, and also scientific basis for each technique, was not familiar to [the mentee].”

In general, mentees expressed gratitude for mentors’ expertise and experience. There was also particular mentee appreciation for mentors’ integration of Indigenous perspectives into research experiences. One mentee said, “the mentors in this program are just wonderful” and most agreed, referring to them as “awesome,” “amazing,” “understanding,” “open,” “welcoming,” “down to earth,” “so nice,” and “very influential.” Mentors and mentees described their relationship as “close,” “caring,” and “encouraging.” One mentee explained, “working with [the mentor] was fantastic, my experience was really good.”

Mentees recognized involvement of people from community organizations, including artists, cultural educators, social workers, and secondary school teachers who partnered with the UNMC-YES program to support mentorship in research education. Mentees and mentors furthermore identified family members as influential in their experiences of health research. One mentee described wanting to follow in their mother’s footsteps, saying, “I feel like I have a bit of an obligation to go as far as I can, kind of like feels like it’s like passing the torch in a way.” One mentor shared this desire to ‘pay it forward’, saying, “I have had a lot of really great mentors throughout my academic journey and professional journey, and so I really hope that I can be that kind of mentor for students, that I’ve had in my experience.”

People representing different Tribal nations, fields of research, communities, schools, and families reported working together to build the AI/AN workforce in health research. In addition to formal research mentors, mentees engaged with UNMC-YES partners in community events that supported their development as Native scholars. In some cases, mentees identified their ancestors, relatives, and Tribal nations as motivation and support for their pursuit of research and many acknowledged the desire to give back to their community. For example, one mentee explained:

I always knew specifically within my tribe, my community and probably a lot more, lung and liver cancer are very prominent, and I always knew that. And so I think that kind of connected me more to this cancer research.

### Settings: An Intentionally Diverse Foundation.

A Native Hawaiian proverb, ʻO ke kahua ma mua, ma hope ke kūkulu, meaning “The site [foundation] first, and then the building,” emphasizes the importance of setting intentions, including purpose, and building upon culture, worldviews, history, place, and time. It was evident that mentors and mentees recognized shared purpose in increasing AI/AN representation in health research careers. One mentor stated, “I think the goal of the program is to get kids that don’t necessarily have access to opportunities to work with researchers.” One mentee explained, “Where I’m from, there’s … no labs, … there’s no research facilities …So it’s it was all new to me… growing up on the reservation, having a lack of resources to pretty much anything.“ Another mentee acknowledged, “I’ve always wanted to be in research, but I just never got the opportunity…It provided me a way to get into research like a foot in the door.” Mentees saw the importance of diverse representation in health research to address health inequities. One mentee elucidated:

It was really hard to get good health care and really the only health care we could get was like Indian health clinics. And I don’t know, I personally always just had a problem with them. I feel like they’re really understaffed and underfunded… If I’m going to an Indian health clinic, I would rather be treated by a doctor that looks like me and knows like kind of my experiences, rather than a White doctor because it’s always a White doctor and then like the receptionist or something is Native.

In describing the UNMC-YES mentorship, one mentor said that it is “an opportunity for us (Native peoples) to shine” and elaborated, “you know, it’s an opportunity for us to grow and learn, and contribute.” Some mentors made an effort to approach the mentorship with cultural relevance, acknowledging AI/AN culture; however, a few non-faculty mentors were not made aware that the YES mentees identified as AI/AN or that the YES program aimed to acknowledge AI/AN culture in research experiences.

Overall, mentors expressed a desire to support under-represented minorities in health research. As one mentor reasoned, “I think as an educator… we all feel passionate about bringing up and training underrepresented, you know, minority students.” Another mentor explained, “it’s always a great opportunity to see them (mentees) leave the lab and actually want to continue in the lab.” Mentees reportedly participated because they perceived the internship to be a “good opportunity” that could “encourage lives of other people and help other people.”

Mentees expressed appreciation for opportunities to share what they were learning by serving as mentors themselves. Commenting on being in the role of a mentor to future generations of AI/AN youth, one mentee said:

There was one (event) that we did and we had all children come up to us and we were working with these kids on different methods of, you know, how they can protect themselves and how to teach them not to smoke and just seeing the joy in the children’s eyes…hands-on doing experiments… I really miss that part of it.

The same mentee commented, “what worked for me is the way I was able to relay the knowledge that I have learned to the children …that has definitely worked for me.” Another mentee discussed “trying to be a role model for people, especially like Native students.” Yet another shared their appreciation for being able to mentor youth in a citizen science pediatric cancer project and teaching youth to test carcinogen levels in waterways. One mentee explained this as an iterative learning cycle, emphasizing how “just like talking to students and helping them learn about what cancer research is. It helped me get a better understanding of it as well.” Mentors and mentees shared knowledge with the goal of building up future generations of AI/AN health researchers through the UNMC-YES program.

Many mentees expressed gratitude for the mentorship, the mentors, and the structure of the program. Mentees described a flexible work commitment to their participation and schedule, which, they reported, allowed them to priori-tize family and other commitments when necessary. In some instances, this flexibility “was really huge for [the mentee];” one described the ability to “re-schedule” as supportive of their ability to participate. Mentors and mentees were overwhelmingly satisfied with their mentorship experience; one mentee stated, “[The mentors] were always respectful, respectful of my time… and they just really wanted the best for me and I could tell that.”

Overall, mentees and mentors described diverse research settings and adequate space and resources for learning opportunities. Mentors emphasized the importance of exposure to “every aspect of sort of biological research or biomedicine and biology, including the research side of things.” Although the program did not require cross-training, half of the mentees gained diverse exposures to research in both biomedical and public health internships. Moreover, exposure to various lab techniques and/or research methodologies helped sustain more long-term mentorships.

The power of the mentorships was made very clear when one mentee said:

[The mentors] *kind of like opened my eyes to what health care could be. It didn’t have to necessarily be something that was so specific, so narrow. It’s actually something that’s very broad. And there’s like a bunch of different things you can do with it, do with within health care. So in a way, they kind of exposed me to some of the different things I could do*.

One mentee explained that participating in the UNMC-YES mentorship “kind of elevated [their] opinion on research, pursuing a research career.”

### Problems: Lack of Contextual Awareness.

Although the data overwhelmingly evidenced very positive experiences for both mentors and mentees, some problems did arise when mentoring settings did not facilitate exploration of contextual factors to ground mentees’ and mentors’ interpretations of their roles and responsibilities. At times, mentors and mentees reported a mutual lack of familiarity with the UNMC-YES program and a lack of awareness of contextual information about each other (identity, scientific background, culture, goals, interests, etc.). Mentees and mentors expressed that there were times when personal situations (e.g., having a baby, class schedules, illnesses, transportation issues, or other jobs) affected their ability to participate in the mentorship and interact with colleagues. Mentors and mentees alike attested to the fact that personal background (e.g., family structure and history, cultural background, and lived experiences) influenced their relationships with their colleagues and their research.

Some mentors and mentees engaged in research experiences without establishing familiarity between each other in terms of culture and research. A few mentors noted that mentees seemed intimidated by them, stressed out by them, or avoided direct contact with them. Moreover, two mentees noted that the individuals whom they identified as their mentors (PIs) were not present, available, or responsive, with one saying, “My mentor …really didn’t come into the lab at all. So [the mentor] had graduate students teaching me.” As one mentor said, “I don’t think we got very close over the whole procedure. I think I honestly made [the mentee] kind of nervous. So, [the mentee] didn’t seem like [the mentee] was super-open to talking to me — with me about things.” More than one mentor recalled not being aware of the mentees’ Native background. Multiple mentees said that they were not certain if their mentors even knew that they identified as Native because their personal background never came up in conversation. A PI mentor commented, “we (the mentee and I) didn’t talk that much” and a postdoc mentor said, “we (the mentee and I) didn’t see each other very often.” Overall, mentors expressed a desire to “know more background information about the trainee,” including future goals and expectations for the internship.

One mentor commented on their lack of awareness of the UNMC-YES program aims, noting, “I don’t know if when I started this program that I was aware it was oriented towards exclusively, like Native American students.” One mentee also described a lack of familiarity with the UNMC-YES program internship structure, stating, “I didn’t exactly know what I was going to be doing, so I guess kind of like setting those expectations would have been a little nice in the beginning.”

The following is a good example of the problems that arose when there was a lack contextual awareness between a mentor and mentee. As described by the mentor, a mentee “had another job” and “treated this internship as a sort of job… So, clock-in/clock-out.” The mentor also saw their relationship as purely professional, and when asked to describe their relationship, stated, “I guess I’m a professor to [the mentee] [laughs]. [The mentee] is an intern to me. What else? I guess, that’s pretty much it.” The mentor described how the mentee “would just pour the cells instead of actually pipetting up,” reasoning that the mentee “was driven by, ‘I need to get this thing done’.” The mentor identified this as a lack of interest in the “academic pursuit” and “some sort of like a learning curve in terms of how we interact in a professional setting, and how science is done, and what science is about.” The mentor also revealed that the mentee “doesn’t really wanna readily engage in interactions with [the mentor] … [the mentee] goes directly to the grad student. [The mentee] has a good relationship with [mentee’s] graduate student… So, maybe [the mentee] is intimidated.” This same mentor shared, “I’m not quite clear what [the mentee’s] goal is through this research program… I haven’t really spoken to [the mentee] explicitly about what [the mentee’s] goal is, in terms of getting this research experience. At some point, I guess, I’ll have to have that talk.” Beyond this, the mentor expressed a lack of knowledge about the UNMC-YES program aims, stating, “I’m not sure what the end point outcome that the program is looking for.” Opportunities to build personal and professional communication between mentor and mentee and/or mentor and UNMC-YES personnel were not structured into the UNMC-YES program. In some situations, the absence of relationships hindered understanding of research team organizational structure and clarification of roles and responsibilities.

One mentor reported that “the biggest hurdle is, kind of, [the mentees’] lack of experience when they start.” While some mentors described being comfortable with meeting mentees where they were at, others described a need for students to have previous research experience, background in science, and/or a major in a science field. This was evidenced in the following mentee report that described a mentor with high expectations:

[The mentor] *wasn’t super responsive to questions or like very really mentoring me. The graduate students who I worked with were amazing while they were there. But once they left, that was kind of like trying to figure things out on my own, which was which was really difficult… I don’t think personally, I need like 24–7 supervision, but there were just some really like. There would be situations where I would have questions and they just weren’t really answered ever. And I wasn’t sure like I would be asked to do a new technique but never shown how to do it. And then there would be frustration when it wasn’t going well. And still, no one would show me how to do it. So I was trying to go based off like a kind of incomplete protocol by myself. And that part was hard… We had to make our own (gels). But the protocol, like when you got to the portion where to make the gel, there wasn’t enough. It just said ‘for appropriate gel’ with no other instructions on what that meant… I had never done that with these like instruments, and it was a different approach. So I was like, I don’t know how to do this. And* [the mentor] *would just tell me, ‘Well, read the protocol’ and that’s what I was doing, the protocol is incomplete, and then* [the mentor] *told me, ‘Well rewrite the protocol so that it is complete.’ I said, ‘I already don’t know how to do it.’ So that was frustrating…I’d be the last person leaving the building, and my gel didn’t work and I’m here till eight o’clock. I just want to cry*.

A mentor’s lack of understanding of this mentee’s knowledge and perspective led to unrealistic expectations of the mentee, which resulted in negative experiences for both the mentee and mentor. As another mentee described, “I would just go in and I would be by myself most of the time, and I would just…they would tell me, like what to do and I would do it, and then I would just have someone, sign off on my time sheet every week. So, there wasn’t a whole lot of interaction that I got from that, from being in the lab.” Another mentee said, “for about a year, like the last half of the year that I worked, there, I was there with almost no one else in the lab.” As one mentor noted, “I feel that [my mentee is] in a little bubble. That [my mentee] is among all these researchers, and there’s pre-meds and all these, like, really aggressive students and …I sometimes feel a little bad, [my mentee] is in a little bubble [by themselves].”

Paralleling mentees’ varying levels of familiarity with science was mentors’ varying levels of familiarity with Indigenous culture. In fact, mentors and mentees engaged in research experiences that, at times, operated under the assumption that Western and Indigenous cultures, values, worldviews, methods, and ethics were mutually exclusive. Mentors and mentees highlighted difficulties in finding ways to integrate Western science with Indigenous science. When asked to describe their role, one mentor said, “I viewed myself as kind of just trying to teach technique and basic lab stuff.” At times, the program’s aim to offer culture-based mentoring was not communicated to mentors. When asked if they acknowledge Native American culture, some mentors clearly answered “no,” others chose not to answer this question, and one explained, “we didn’t do anything that focused on that (culture).” One mentee reasoned, “We haven’t really touched cultural subjects like organically, just because of the nature of how our lab works…we’re kind of like working on stuff …we haven’t really had a, I guess had a chance to really talk about like our cultural backgrounds.” Some mentees did not recognize ways that their culture could factor into their work and many could not identify ways that mentors fostered their Indigenous identity through the research experience. Most mentors had little familiarity with AI/AN culture or Indigenous research methods and the majority reported no prior experience in mentoring AI/AN students. Although Western and Indigenous perspectives, practices and pedagogies can complement one another, mentors and mentees were not familiar with the program’s strategies to promote the integration of the two. As one mentor explained:

*…it might be a cultural aspect, I’m not really sure, but they are different interns…a YES mentee, can be completely different than undergrad mentees that I’m used to. So, had I known that from the beginning, I would have had different ways of training* [the mentee] *rather than putting* [the mentee] *through the regular undergrad training method*.

### Actions: Working Together.

The word gadugi, in Cherokee, meaning “people coming together as one and working to help one another,” reflects the actions of both mentors and mentees. We found that mentors’ and mentees’ misconceptions of the mentorship purposes and processes that came from a lack of contextual awareness was often addressed in their actions, in working together. Sharing in the practice of research together created opportunities for mentors and mentees to connect and not only learn about the scientific process but also learn about each other and the UNMC-YES program to build context.

Mentees participating in biomedical research detailed a spectrum of lab techniques and hands-on cancer education experiences as significant components to their experience. Mentees reportedly performed a variety of techniques including western blotting, immunofluorescence, cell culture, and transfection. One mentee explained, “I just kind of like pipette a lot of stuff all day …. I kind of just culture cells in a petri dish.” Another mentee explained that prior to the internship they “had no clue at western blotting,” and recounted, “I have definitely learned new things from this internship.” One mentee who worked in labs at two different universities experienced two different approaches to western blotting:

*I came to* [2nd University] *and they had a different setup, like a different approach. They did a dry transfer, which I had never done before and at* [1st University] *we had always used pre-made gels and at* [2nd University] *we had to make our own*.

Another mentee shared, “I’ve learned a lot…the biggest thing I’ve learned is just like how research is conducted and then like medical ethics.”

Mentees participating in public health research detailed a community-based approach to addressing cancer through conducting community readiness assessments, scoping reviews, or art-based research. One mentee described:

I did interviews with a cancer patient. They had every single kind of skin cancer that you can imagine. So I did eight interviews, personal interviews… I researched the subject matter of all kinds of cancers and wrote reports on it. And — and then I did arts-based research where I did drawings…and paintings related to the…to the cancer itself.

Some learned about Indigenous culture through traditional methods of knowledge generation such as artmaking, storytelling, and talking circles (a traditional Native American form of reflective group communication where individuals have an opportunity to speak without interruption). One mentee described a mentor “that always brings the Native spirit into it,” noting, “every meeting that we have, we always do a Native poem or somehow we bring the Native culture always into what we’re learning,” and shared that this was “definitely a great aspect.” One mentee described that through UNMC-YES they “really [got] connected, you know, in the Indigenous community.” Mentees and mentors participated in various outreach events (e.g., tabling booths at powwows, teaching science lessons in schools, presenting workshops at community gatherings, and exhibiting research at art shows and local health fairs) with the AI/AN community. In addition, mentors helped connect mentees to local cultural experts, artists, health care professionals, and other community members who supported the mentees in unique and individualized ways. As one mentee expressed:

I would say just inviting the cultural specialists in was definitely something that, you know, putting it emphasis on our identity as Native people just like, you know, not let us forget that we are Native that was something that was very important.

Some mentees described gaining research experience in both biomedical and public health settings:

I’ve done multiple things. So for my first project, I was in a lab doing lab work and assisting with research in that way. And then my second one, it was more community-based research. So it was like interviews and recordings and going through transcripts and then helping write.

Mentees’ research interests and career choices were influenced by the exposures they had through mentorship. As one mentee explained, “I was interested in going to medical school, but now I’m leaning more towards biological research.” This mentee elaborated:

[The mentor] *just seemed so passionate about it and like* [the mentor] *was so excited to share about it and the way* [the mentor] *explained the information* [the mentor] *had and would be like and then next week, I’m going to teach you about this, you know, all excited, and it just really made me think, Hmm*, [the mentor] *can be passionate about this*. [The mentor is] *so passionate about it. Maybe, maybe I would be passionate about it or enjoy it*.

Another mentee made clear, “you have mentors so your mentors can help guide you into what field … that you need to go to.” Some mentors were engaged in guiding mentees’ self-exploration as Native scientists and scholars. As one mentor stated:

[The mentees’] *eyes have been opened to the world of, of research and how, how vast and diverse and how much opportunity there is out there to be involved in research… I think they’ve also learned what they bring to the table and how they can specifically contribute. So, what their strengths are and – I think overall, they’ve just kind of learned more about themselves and what their interests are, what they’re able to do, what they want to do, and how that can – how that works with who they are, and their cultural background*.

Mentors described the development of mentees’ expertise in health research “in terms of their understanding of what research is, in that you devise questions, that you perform techniques, that you confer with other people.” One mentor described how “[the mentee] was able to come up with [the mentee’s] own projects for a while and kind of, direct those under [the mentors’] supervision,” “[the mentee] was able to read scientific papers and kind of develop ideas from that” and “[the mentee] was actually able to participate in manuscripts that we’ve submitted.” Multiple mentees reported attending conferences with mentors not only as an audience member but also as a presenter. Manuscript preparation and authorship were also commonly described by both mentees and mentors as a part of the research experience. Proficiency in lab technique was also described in one mentor’s statement: “[the mentee] did a lot of, I think, like western blots and stuff, so [the mentee is] pretty proficient in those.” Additional examples of mentor and mentee activities are provided in Table 2.

### Resolutions: Balancing the Importance of Knowing Ourselves, Each Other, and Our Situation.

The solution in this story aligns with the Diné philosophy of Hózhó, the pervasive harmony of all things and the universal task for us to emulate this balance within ourselves and the situations we are presented to ensure what we do is meaningful and successful. Striving to create supportive and positive relationships that foster a connection between the research and the research context can help address the problems described by participants.

Mentorships were described most positively when mentors and mentees expressed mutual familiarity with one another (both personally and professionally) and with the UNMC-YES program (background and aims). Mentors’ and mentees’ familiarity with one another was unmistakable in some instances. For example, one mentor fondly remembered being asked to be a bridesmaid in the wedding of one mentee. Moreover, mentees’ descriptions of the mentorship as “flexible,” “adaptable,” and “accommodating” suggested contextual awareness. Some mentees noted that “there’s not a set schedule” and explained, “it’s very flexible which is something I really need.” In addition to flexibility in scheduling, mentors and mentees reported adaptability in settings (e.g., transitioning from a lab-based internship to public health) and in their scope of work (e.g., giving mentees options in task assignments).

In success stories, mentor and mentee relationships were at the heart of the mentorship. One mentee explained, “[the mentor] was like very encouraging and showed me how to do things, was very supportive.” The majority of mentor/mentee relationships were described as “open,” “friendly,” and “positive.” Some mentees described the mentorship as increasing their confidence in pursuing health research. “[The mentors] definitely have impacted me — just supporting…or they help me believe in myself that I can do it,” said one mentee. Another mentee said, “Sometimes I feel like I’ll be having like a really hard time. We’re like, Oh, kind of get on about myself. And, then like, I’ll talk to like one of the mentors or something… They make me feel appreciated.”

Mentors responded quite similarly to mentees when asked to describe their relationships with mentees. One mentor expressed their hope for the mentee to see them as a “colleague” or a “source of support in the future.” Another mentor described relationships with mentees as, “positive, uplifting, encouraging, trusting.” Similar to the mentees, some mentors described that they were also “growing and learning” from the research mentees and also reported encouraging personal relationships with the students. As one mentor explained, “I’m very fond of them (my mentees).And I always will be.” When describing their accomplishments, this mentor noted, “Both of them (my mentees) had a big interest in helping people… I really value that.” One mentor described wanting to “be that person that they (mentees) can be vulnerable with, that they (mentees) can come to with questions, that they (mentees) can share their experiences with and share cultural perspectives with too.”

One mentee commented, the mentorship “made me feel like I am pretty confident in knowing that this is what I want to do and like this is who I am.” Another mentee said:

I think that was something that was very important. You know, just being able to talk about my culture, you talk about like some things I could do couldn’t do within research. So kind of finding a healthy balance helped where I wasn’t compromising myself, who I was with the research I was doing. So finding a way that they could also tied it tied it back to home too it was also very important.

One of the mentors requested more opportunities for mentees and mentors to collaborate and get to know each other, saying:

It’d be so nice if there was some sense of a cohort. You know, like we’re part of this together, not only could the mentors come together and talk about what they’re doing and support each other, but also the, the mentees. So the interns could get to know each other from other areas… to build real friendship, and understanding, and trusting relationships.

One mentor noted:

[The mentees] *were all interested in connecting and learning more about who they are and how that relates to their studies and the work that they do. So, our Native culture and, and, and Native scholarship, can support what they’re doing and the way that we see and do, and understand things*.

A strong personal and professional relationship among mentors and mentees supported tailoring research projects to the mentee’s/mentor’s background, interests, and goals. Mentees described forming personal and trusting relationships with mentors; one explained, “it’s just a strong — I think it’s a professional relationship and an academic relationship, but it’s also personal too.” Mentees appreciated the opportunity to discuss personal topics with mentors such as, “being able to talk about home,” “how hard college can be,” or “health issues.” One mentee said, “[the mentor has] been basically kind of like a friend in a way. So, I’m glad to have that relationship with [the mentor], and it’s like that with all of my other lab mates in my specific lab.”

Building trusting relationships between mentors and mentees involved a certain amount of cultural humility. Most mentors were not familiar with Indigenous understandings of science. However, as one mentee explained, their mentor “understood where [they] were coming from” and “that’s what drew [the mentee] to this program because [the mentee] was like, these people know where I’m coming from and they understand.” Another described their mentor, saying, “they aren’t specifically Indigenous, but they come from the respect of being Indigenous.” One mentor described efforts to “create new pathways and break molds or…decolonize academia in some ways.” Some mentors described a desire to make the mentorship more culturally relevant to the AI/AN students. One mentor noted,

I think there are things over time that I can learn from others in our program… For example, about whether there are additional ways of instruction related to lab science from an Indigenous perspective. I think it’s more obvious to me what that means from the public health side and the clinical side. I have less understanding of what that may mean from the lab side.

More understanding of where the UNMC-YES program was coming from was supported by conversations about the big picture: how lab personnel were organized, how specific lab techniques performed related to the overall study design or how the research questions related to the AI/AN community. One mentee said, “there was a lot of emphasis on Native, like Native approaches to science.” An example of cultural humility was shared in the following description of a mentor by a mentee:

[The mentor is] *very understanding. I feel like* [the mentor is] *very educated, which I feel like when you talk to like, like, like non-Indigenous or* [non-] *Native people, it’s really hard to find people that are so like educated. I feel like within the YES program, I see all the time like they, they kind of all are very understanding of boundaries like respectful of culture and just know a lot about it*.

Some mentors encouraged mentees to learn more about who they are and where they come from, and to include that in writing biosketches or biographies. An emphasis on researcher positionality supported mentee development of research ethics and helped inform the ways in which research interns conducted, analyzed and disseminated research. One mentor described working to ensure that “it’s (the mentorship is) all somehow related to who they (mentees) are and who they (mentees) wanna serve and, and why that’s important to them.” Another discussed “helping [the mentee] to understand the bigger picture of what it means to be a researcher, and how to develop yourself, in a way that will help you achieve those long-term goals, whatever those long-terms may be.” This was evident in one mentee’s comment, “I’ve always wanted to be a healer and so that for me is rooted in like my cultural background.” Some mentees reported writing biographical statements, which helped establish their positionality.

Acknowledging and locating one’s views, values, and beliefs in relation to the research process proved to benefit both mentors and mentees and allowed them to better relate to one another. As one mentor explained, “It’s really awesome to have – to be surrounded by, by peers, colleagues, students that understand you and are aligned with your ways of being, and knowing, and doing.” Overall, mentors’ and mentees’ awareness of each other’s varying backgrounds, levels of experience, expertise, and comfort related to research, Indigenous perspectives in science, and the UNMC-YES mentorship program facilitated successful mentorships.

## DISCUSSION

There is a critical need to increase the representation of AI/AN students pursuing health research professions (AAMC and Association of American Indian Physicians, 2018; National Science Foundation, 2019; WGDBRW and ACD, 2012). Any efforts to do so must address the history of abuse in research and a lack of cultural awareness in academic health institutions (Hodge, 2012; Stiffarm and Lane, 1992; Thornton, 1987). Scholars in this field note that this work requires collaborative efforts between AI/ANs and non-AI/ANs to embrace Indigenous worldviews and support culturally responsive research methods (Burgess et al., 2020; Walters and Simoni, 2009; Walters et al., 2019).

Health research mentorships have the potential to facilitate such collaboration. However, little is known about the complexities of the roles of mentors and mentees in Indigenous health research mentorships. This study was the first to investigate Indigenous and non-Indigenous mentor experiences alongside Indigenous mentee experiences. It is clear that there is no one-size-fits-all mentorship model, or any universal strategy designed to support the diverse needs and success of AI/AN research mentees in health sciences. However, lessons learned from this story can help guide both mentors and mentees in mentorship practices.

Through collective re-storying, we explained mentor and mentee perspectives of lived experiences in the UNMC-YES mentorship program. Although some scholars call for a mentee-centered approach when mentoring Indigenous students (Murry et al. 2021), we recognized a need to center the relationship between both mentee and mentor, which is supported by multiple scholars who have found that viewing mentorship as circular rather than hierarchical can foster multi-directional learning that can build on the strengths and weaknesses of both the mentor and mentee (Ferguson et al., 2021; Halas et al., 2017; Mullen and Cox, 1997).

The novelty of this study is the appreciation for the roles of both mentees and mentors in mentorship. This aligns with Liaw and colleagues’ (2016) emphasis on culturally effective mentoring relationships, which they explain requires a commitment to building mutual trust, maintaining respect and dialogue, recognizing identity and positionality in research, leading and advocating for the community, having knowledge of health systems and services available to the local community, and effectively communicating cultural knowledge. A mutual approach to mentorship can facilitate opportunities for culturally effective health research mentoring for AI/ANs.

When mentors and/or mentees lacked contextual awareness with regard to the UNMC-YES program, problems arose. Notable was one mentee’s perspective that the graduate student they worked with was not formally in the role of a mentor. Disparate understanding of the term “mentor” could have contributed to uncertainty and lack of mutual understanding. Depending on the size of a faculty member’s research group and the many duties assigned to faculty members, in the UNMC-YES program, a faculty member might have little or no time available for directly teaching, helping, or advising an intern, but still have graduate students or postdoctoral associates who are able to assume those functions well. Considering this in program design, mentorship orientation, and in the use of the term in evaluations of the program will be advantageous.

Noteworthy is the role that program personnel can play in structuring mentorships and informing mentors and mentees about their roles and responsibilities. In this case, the absence of mentor emphasis on AI/AN culture was due to the absence of UNMC-YES program leadership in that regard. Also worth mentioning is how Elders and other community-based educators who integrated Indigenous culture, pedagogies, and research methods were identified by mentees as “mentors.” The fact that only one of the eight mentors identified as Indigenous not only corroborates the need for more AI/AN health researchers but also demonstrates a need to collaborate with AI/AN community members and support non-Indigenous mentors with training and resources to implement culture-based approaches to research and mentorship.

AI/AN faculty find it challenging to provide adequate support for AI/AN students because they face racism, discrimination and the “minority tax,” which is an obligation to take full responsibility for diversifying academic medicine (Rodriguez et al., 2015; Tippeconnic Fox, 2005). There is, thus, a need not only to recruit but also train non-AI/AN faculty as mentors to AI/AN students. Scholarly literature on mentoring Indigenous students recognizes that most mentors are not Indigenous themselves and acknowledges a need for ongoing mentor training that addresses diversity and moves beyond cultural competency toward cultural humility and safety (Walters et al., 2016).

Mentees and mentors described what has been referred to as “walking in two worlds,” a metaphor that represents the significant and invisible challenges experienced when reconciling the often conflicting values and ethics of Indigenous worldviews with the values of dominant, colonial society (Styres et al., 2010). At times, participants described a divide between mentors and mentees, biomedicine and public health, Indigenous and Western worldviews, personal and professional activities, cultural and scientific practices. In mentorships involving Indigenous mentees, it is important for mentors and mentees to share their worldviews to facilitate effective mentorships. It is our hope that this story will support mentors and mentees in that process, as they both walk in two worlds, together acknowledging each other’s diverse backgrounds, knowledge, interests, strengths, and challenges.

Through collective re-storying, we present all experiences as opportunities to recognize where/how the UNMC-YES program and Indigenous health mentorship can be strengthened in general. We offer the following recommendations to facilitate more contextual awareness for both Indigenous mentees and their mentors in mentorship programs:

1. Engage both mentors and mentees in AI/AN community events, trainings, and coalitions.
2. Develop a structured mentorship protocol in consultation with mentors, mentees, Elders, cultural educators, AI/AN educational partners, and program personnel.
3. Strengthen program orientations by including mentor and mentee introductions to all program personnel, program protocol, and program-specific goals.
4. Identify and integrate mentor and mentee strengths by establishing positionality and setting goals that acknowledge the mutual objective of supporting AI/AN students and their communities.
5. Structure the program to include regular meetings between mentors and mentees and ongoing talking circles or opportunities for program personnel, mentors, and mentees to build meaningful and supportive relationships.
6. Regularly request formative feedback from participants (i.e., check-in with mentors and mentees about their experiences) to support program evaluation and solve problems in real time.
7. Require mentor and mentee cultural training with an introduction to both Indigenous and Western perspectives of the history of health research as well as both Indigenous and Western science, pedagogies and research methods.

The UNMC-YES cancer research internship is targeting these strategies to improve program delivery and quality for mentees and mentors alike. These findings can also support and inform the development, evaluation and maintenance of other health research mentorship programs for Indigenous students.

### Limitations.

UNMC-YES program implementation varied greatly across the program sites and across mentorships. Sampling proportional to the representation of mentees and mentors across sites was employed to ensure that these diverse experiences were reflected in study findings. The use of purposive sampling, nonetheless, could present selection bias. Although we tried to control for response bias by ensuring that each mentee was interviewed by someone other than their mentor(s), mentee responses could have been influenced by their relationship to individuals within the research team and/or their relationship to the UNMC-YES program. The majority of UNMC-YES program mentors were biomedical researchers and persons who did not identify as Indigenous. The research team, however, was comprised of more public health researchers and persons who identified as Indigenous. It is important to note the composition of the analysis team as all members have studied Indigenous research methods and value Indigenous perspectives and pedagogies. Positionality of these researchers influenced the research design, the type of data collected, and the way that the data were analyzed.

The findings of this study can support successful future AI/AN mentorship in cancer research and, more broadly, health science or STEM research. Additional studies, including Indigenous scholars and Indigenous methods, should be conducted to evaluate our recommendations for effectiveness in successfully retaining and encouraging the matriculation of AI/AN students into health professions and research fields.

## Figures and Tables

**Table 1.** Organization of story elements in a problem-solution narrative structure using collective re-storying analytical approach.

Story Element	Definitions	Themes
**Characters**	Persons in the story: their role, personality, background, attitude, style and perspectives	A Diverse Circle of Relatives
**Settings**	Context in the story: history, culture, place, time and purpose	An Intentionally Diverse Foundation
**Problems**	Issues in the story or phenomena that negatively affect the persons and need to be addressed	Lack of Contextual Awareness
**Actions**	Events in the story that illustrate persons’ thoughts, intentions, feelings and behaviors	Working Together
**Resolutions**	Solutions in the story that support adaptation or transformation that leads to positive outcomes and resolution	Balancing the Importance of Knowing Ourselves, Each Other, and Our Situation

Note: Definitions adapted from Ollerenshaw and Creswell (2002).

**Table 2.** Examples of mentor and mentee activities.

Participant	Example Quote
**Mentor**	“[The mentee] was able to witness some of the seminar preparation, and then able to run like DNA gels and kind of analyze those.” “I think [the mentee] learned … some new techniques, like the extraction or some genotyping. And also understand some mouse model creation strategies… got to know some like basic cell culture techniques…” “[The mentee] did a lot of, I think, like western blots and stuff, so [the mentee is] pretty proficient in those.” “[The mentee] gained that professional attitude towards her colleagues, as well.”
**Mentee**	“[The mentor] did… resumes and stuff and like bios-was helping people work over that.” “[The mentors] always say, just be- just know you can ask questions and they ask sometimes periodically through the Zoom presentation if we have any questions. And even afterwards, so they- they really want you to ask questions. There’s no shame in asking them.” “I learned a lot about cancer and cancer research. I attended some of the lunch seminars that were held at the [institution]…” “[The mentor] always shared with me anything that [the mentor] gets that’s centered around Native Americans. And so [the mentor] recently shared with me a conference for Native Americans, I think, cancer treatment, a group or initiative or something like that.”

## ABBREVIATIONS

AAMC: Association of American of Medical Colleges
AI: American Indian
AN: Alaska Native
IHS: Indian Health Service
IRB: Institutional Review Board
MPIs: UNMC-YES Principal Investigators
NCI: National Cancer Institute
STEM: Science, Technology, Engineering, or Mathematics
UNMC: University of Nebraska Medical Center
YES: Youth Enjoy Science
